# Two reasons to kill: predation and kin discrimination in myxobacteria

**DOI:** 10.1099/mic.0.001372

**Published:** 2023-07-26

**Authors:** Christine Kaimer, Michael L. Weltzer, Daniel Wall

**Affiliations:** ^1^​ Department of Biology and Biotechnology, Ruhr University Bochum, Bochum, Germany; ^2^​ Department of Molecular Biology, University of Wyoming, Laramie, WY, USA

**Keywords:** *Myxococcus xanthus*, outer member exchange, rearrangement hotspot, tight adherence transport system, type III secretion system, type VI secretion system

## Abstract

Myxobacteria are social microbial predators that use cell–cell contacts to identify bacterial or fungal prey and to differentiate kin relatives to initiate cellular responses. For prey killing, they assemble Tad-like and type III-like secretion systems at contact sites. For kin discrimination (KD), they assemble outer membrane exchange complexes composed of the TraA and TraB receptors at contacts sites. A type VI secretion system and Rhs proteins also mediate KD. Following cellular recognition, these systems deliver appropriate effectors into target cells. For prey, this leads to cell death and lysis for nutrient consumption by myxobacteria. In KD, a panel of effectors are delivered, and if adjacent cells are clonal cells, resistance ensues because they express a cognate panel of immunity factors; while nonkin lack complete immunity and are intoxicated. This review compares and contrasts recent findings from these systems in myxobacteria.

## Introduction

Conflict systems are widespread among bacteria and often result in a cell death response [1, 2]. These systems serve different roles, including nutrient acquisition, self-defence, and ensuring populations are homogenous and cooperative. In some cases, conflict systems behave altruistically, such as when a phage attack triggers a programmed cell death response, which prevents phage propagation and sibling infection. Other conflict systems kill competitors to promote individual fitness. In this review, we focus on a subset of systems used by myxobacteria for predation and kin discrimination (KD).

**Fig. 1.**
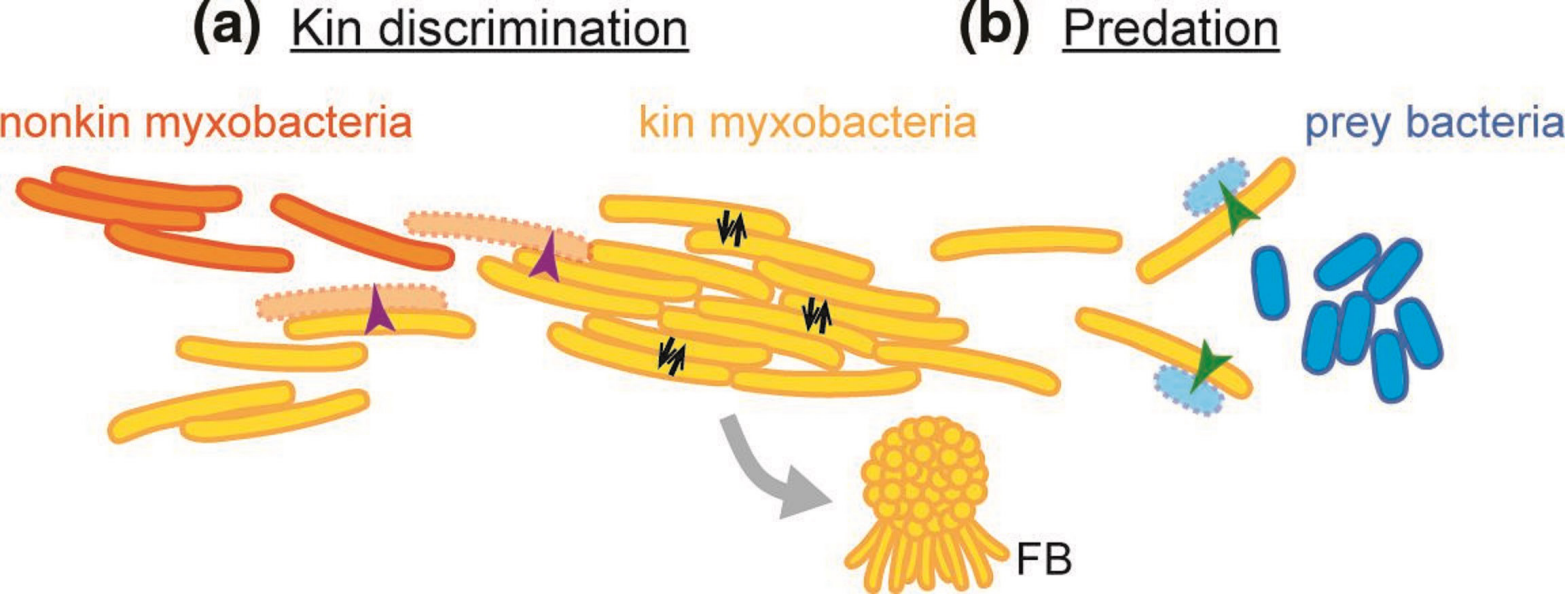
Conflict systems in the myxobacterial life cycle. (**a**) KD allows myxobacteria to engage in costly social behaviours only with their closest kin (yellow), which ensures congruent vegetative populations and homogenous developmental fruiting bodies (FB). Nonkin myxobacteria (orange) are recognized and eliminated (purple arrows). Within a kin group, OME (black arrows) can benefit the fitness of a population. (**b**) Predation serves the acquisition of nutrients, as myxobacteria specifically kill nonkin cells of other species (blue) and feed on the released biomass. Part of the multilayered predation strategy is cell-contact-dependent prey killing, where a single myxobacterium kills and lyses a prey bacterium in a cell-contact-dependent manner (green arrows).

Myxobacteria are ubiquitous soil-dwelling microbes that combine both single-cell and multicellular behaviours, enabling them to maintain a complex social lifestyle (Fig. 1) [3]. Single cells glide on surfaces with the aid of lateral motors, but they also frequently assemble into multicellular swarms that move coordinately, primarily driven by type IV pili-dependent social motility. Multicellular swarms are believed to be involved in social feeding because myxobacteria secrete costly hydrolytic enzymes to externally digest biopolymers; and in groups, cells share this metabolic burden by providing a division of public good production [4, 5]. Rippling is another motile social behaviour in which waves of cells move rhythmically back and forth, apparently to improve predation efficiency [6]. However, the best-described social behaviour of myxobacteria is arguably fruiting body development. In response to starvation, thousands of cells aggregate from their surroundings to construct cooperative multicellular fruiting bodies, wherein cells differentiate into different cell types including environmentally resistant spores [3]. By making this transition, myxobacteria benefit from both single-cell and multicellular lifestyles, as the latter provides erect macroscopic structures that are thought to facilitate spore dispersion and division of labour by differentiation into different cell types. Many myxobacteria species use specific conflict systems for nutrient acquisition by predation or for KD, the latter of which allows congruent assembly of multicellular populations to ensure spores belong to the same social group. As outlined here, these conflict systems use different delivery mechanisms to trigger cell death in target cells.

## Predation: killing others for nutrients

Predatory bacteria of different phyla live in terrestrial and aquatic habitats and have developed various strategies to kill, disintegrate and consume cells of other species [7, 8]. Myxobacteria, and especially understudied families such as the *Haliangiaceae *, constitute a dominant fraction of micropredators in soil environments and might strongly affect the composition of soil microbiomes [9]. For this reason, they were recently proposed to be classified as ‘keystone taxa.’ Notably, predation behaviour is facultative and myxobacteria also obtain nutrients saprophytically from decaying organic material. So far, killing and consumption of micro-organisms has been observed for the majority of described myxobacteria species throughout all suborders, with only few non-predatory exceptions [4, 10].

Generally, myxobacteria are epibiotic predators, as they kill and lyse prey cells from the outside. The biomass released from micro-organisms is rich in amino acids and lipids, which are their primary sources of carbon and energy. Furthermore, purines and pyrimidines released from prey nucleic acids are incorporated via salvage pathways [11, 12]. Predatory myxobacteria are considered generalists, as they kill and lyse various Gram-positive and Gram-negative bacteria, including plant and human pathogens, but they also feed on eukaryotic fungi [5, 13–15]. This broad spectrum of structurally different prey is made possible by a multilayered predation strategy with different predation mechanisms that vary in their efficacy and specificity, and that are predominant in single cells or in multicellular swarms, respectively.

## Cell-contact-dependent prey killing

Single myxobacteria kill bacterial prey cells one-on-one in a contact-dependent manner: when, for example, a single *Myxococcus xanthus* cell approaches a prey cell using gliding motility, it stops when in direct cell contact and induces prey cell death within 5 to 10 min [16–18]. In most cases, the *M. xanthus* cell then moves on to kill the next prey cell, leaving behind the prey biomass for consumption by fellow members of its population [18]. While this behaviour was observed decades ago [19], the molecular mechanisms were revealed only recently. Notably, contact-dependent killing involves the combined action of two protein secretion systems, which are specialized for their function in predation: a Tad-like secretion apparatus and a type III-like system (T3SS*) [16, 17].

**Fig. 2.**
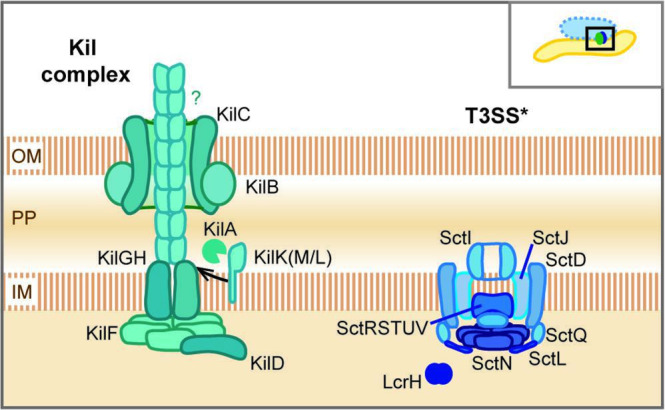
Protein secretion systems that act during predation. The Kil complex is an envelope-spanning protein complex and similar to systems of the Tad (tight adherence) family. Kil is required to induce cell death of prey bacteria, although the putative toxic effectors are unknown. T3SS* is an atypical type III secretion system, as it lacks any OM components and a needle structure. T3SS* is required to initiate prey cell lysis and functionally interacts with the Kil complex in an unknown manner. Both systems accumulate at the predator–prey contact site. See the main text for details on the individual components. PP, Periplasm; IM, inner membrane.

The *M. xanthus* Tad apparatus, which was also termed the ‘Kil complex,’ is instrumental in inducing prey cell death, presumably by attaching to prey and/or delivering toxic effectors; however, the molecular details of these processes and how they recognize/attach to prey cells are not known (Fig. 2) [16, 17]. Kil proteins are encoded in two separate gene clusters, MXAN_3102–3108 and MXAN_4648–4661. Similar to Tad systems of other species, the putative Kil complex spans the *M. xanthus* cell envelope with an inner membrane platform (KilG and KilH) and an outer membrane secretin (KilC), associated with KilB. Additional components are major and minor pilins (KilK, KilL, KilM), a pre-pilin peptidase (KilA) and a cytoplasmic ATPase (KilF). The functions of all remaining proteins encoded within the Kil gene clusters are unknown but, notably, several genes encode fork-head associated domains (FHA), which often mediate signalling processes. KilD, one of the FHA-domain proteins, is essential for prey killing [16].

Tad (tight adherence) secretion machineries are mechanistically related to type II secretion systems, and in many bacteria they produce type IVc pili for surface attachment and biofilm stabilization [20, 21]. In several *Bdellovibrio* species, which are endobiotic predators that invade the periplasm of Gram-negative bacteria, the *tad* locus and the pilin PilA were shown to be involved in prey cell attachment [22–24]. It is, therefore, tempting to speculate that *M. xanthus* attaches to prey cells via a Kil-dependent filamentous structure, but the formation of such a filament has not yet been experimentally verified, and while polar type IV pili are essential for social motility, they are not required for cell-contact-dependent prey killing [16]. Whether Kil functions in the secretion of toxic effectors, either directly or in conjunction with T3SS*, remains to be investigated.

The second protein secretion system involved in cell-contact-dependent prey killing is an incomplete type III secretion system (T3SS*), which shows important differences to the canonical T3SS/injectisome of pathogenic bacteria [17, 25, 26]. The respective gene cluster (MXAN_2434–2464) contains protein components to form an inner membrane platform (SctD, SctJ, SctI) and C-ring (SctQ), the export apparatus (SctRSTUV) and ATPase complex (SctN and SctL), as well as several homologues to the LcrH chaperone (Fig. 2). Interestingly, the *M. xanthus* T3SS* gene cluster lacks a secretin that would span the outer membrane (OM), and it does not contain homologues to a T3SS needle or a translocon structure, raising the question how putative protein substrates are translocated by this system. From an evolutionary point of view, the needle-less T3SS* complex possibly represents an intermediate between the flagellar T3SS and the injectisome and is conserved within the myxobacteria [26, 27]. This argues for a functional, rather than a degenerate system, which has been specifically adapted to act during bacterial predation.

T3SS* mutants are still able to induce prey cell death; however, on a slower time scale than wild-type *M. xanthus *. Notably, *Escherichia coli* cells killed by T3SS* mutants do not lyse, but appear to retain their overall structure, suggesting that T3SS* initiates the disintegration of prey cells [17]. While putative T3SS* effectors are unknown at this point, this hypothesis is corroborated by the fact that *M. xanthus* populations of T3SS* mutants cannot efficiently utilize prey biomass to maintain swarm expansion.

Microscopy analysis revealed that components of the Kil and T3SS* complexes specifically accumulate at the *M. xanthus */prey contact site shortly before the prey cell dies [16, 17]. This hints at a functional interplay of the Kil and T3SS* secretion systems during prey cell killing, but its nature and molecular details need further investigation. Possibilities include the sequential delivery of killing and lytic effectors via a mixed Kil/T3SS* secretion complex, or separate but overlapping functions in prey attachment and secretion of killing factors.

Interestingly, contact-dependent killing mechanisms discriminate between kin and prey cells: only contact with a prey cell, but not with other *M. xanthus* cells, causes accumulation of the Kil/T3SS* machinery and cell death [16, 17, C. Kaimer, unpublished observations]. This implies that the Kil/T3SS* machinery, directly or indirectly, distinguishes self from non-self-interacting cells by a currently unknown mechanism, which may or may not overlap with the KD processes described below. Both Kil and T3SS* were shown to act on different bacterial species, although killing and lysis of fungi or other eukaryotes has not yet been tested [16, 17]. However, while Kil is equally required for predation of Gram-negative and Gram-positive bacteria, T3SS* seems to be expendable when preying on Gram-positives [17]. It is likely that here the lytic contribution of T3SS* can be compensated by other systems that independently secrete a bacteriolytic enzyme cocktail, as discussed below.

Mutation of either the Kil or T3SS* system leaves residual predation activity, but in the absence of both systems *M. xanthus* is unable to move into and lyse bacterial prey colonies [17]. This observation emphasizes the critical role of cell–cell contact by single predator cells, which presumably start killing individual prey cells and make way for multicellular swarms, also described as ‘wolf packs’ (see below) to enter and release high concentrations of bacteriolytic proteins and antibiotics.

## Secreted predation factors

The described Kil/T3SS* machinery presumably translocates toxic effectors at contact sites onto or into prey cells and acts in response to an unknown, cell-contact-dependent recognition mechanism. However, myxobacteria also constitutively secrete a multitude of hydrolytic enzymes in a non-targeted manner and independently of Kil/T3SS*, including proteases, lipidases, nucleases, amidases and glycoside hydrolases, which externally degrade biopolymers [3, 10, 17]. During predation, the main function of these secreted enzymes presumably lies in processing biomass from dead prey, and thereby overlaps with a saprophytic lifestyle. Nevertheless, killing and lysis of live prey by secreted proteins isolated from *M. xanthus* can be observed at high concentrations, and Gram-positive bacteria and fungi with an exposed cell wall are lysed more efficiently than Gram-negative bacteria with a protective OM [28]. A considerable fraction of bacteriolytic enzymes and antibiotics are secreted as cargo of lipid vesicles that emerge from the *M. xanthus* OM [29–31]. After being released from the *M. xanthus* cell, outer membrane vesicles (OMVs) are thought to fuse with the membranes of target cells to deliver their toxic contents [30, 32]. Both bacteriolytic and growth-inhibiting activities have been reported for OMVs isolated from *M. xanthus *, but their efficacy appears to be low compared to other killing mechanisms and might be limited to a few prey species [28, 30, 31].

The bacteriolytic activity of myxobacteria culture supernatants was demonstrated years ago [3, 33]; however, few individual enzymes have since been isolated and characterized. The ones that are include a family 19 glucoside hydrolase, LlpM, which is secreted by *M. xanthus* and targets the bacterial peptidoglycan cell wall similar to lysozyme. It is, however, not strictly required for growth on prey bacteria, and its activity against fungi has not been tested [28]. MepA, a M63 metalloprotease, is enriched in OMVs of *M. xanthus* and contributes to extracellular proteolytic activity [29]. Enzymes with glucanase activity play an important role, especially in fungi and oomycetes predation, with the membrane-associated 1,6-β-glucanase GluM from *Corallococcus* sp. and secreted 1,3-β-glucanase from *Archangium* sp. as recently characterized examples [15, 34].

Secondary metabolites with fungicidal, bactericidal or “toxic” activity on ciliates are produced by myxobacteria in great variety [35–37], but relatively few compounds have been investigated specifically in the context of predation. Here, it is difficult to differentiate whether the predominant function of an antibiotic lies in killing prey for consumption, or in inhibiting the growth of competitors, a strategy also used by non-predatory bacteria. Myxovirescin A (also known as TA) inhibits lipoprotein maturation and causes the formation of lethal cross-links between the membrane and cell wall. Due to this mode of action, the bactericidal activity of myxovirescin A is limited to growing bacteria, suggesting its main function lies in minimizing prey growth in the presence of *M. xanthus* [38, 39]. Some prey species, such as *Bacillus licheniformis *, survive *M. xanthus* encounters specifically by modifying myxovirescin A [40]. Myxoprincomide, a peptide antibiotic with an unknown mode of action, is also involved in predation, but acts solely on Gram-positive bacteria [41, 42].

## Benefits of social predation

Above, we discussed the importance of single, scouting cells for predation. However, the majority of a preying population is engaged in social interactions within a swarm. In an early study by Rosenberg *et al.*, it was shown that saprophytic growth of *M. xanthus* on casein in liquid culture requires a high cell density, which suggested that high concentrations of secreted digestive enzymes are a prerequisite for nutrient acquisition [4]. Also during predatory growth, secreted enzymes, antibiotics and OMVs can accumulate to high concentrations, which theoretically increases the predation efficiency of myxobacteria swarms over that of single cells. This hypothesis has prompted the comparison of preying myxobacteria to wolf packs, where many individuals cooperate and benefit from a shared pool of public goods, which in the case of myxobacteria consists of secreted predation factors; thus, making local prey biomass more accessible for consumption [3, 8, 43]. In support of this, enhanced predation was found in strain mixing experiments. Here, three isogenic *M. xanthus* strains that secreted different 1,3-β-glucanases, produced a cooperative cocktail of enzymes for prey consumption [44]. Nevertheless, the wolf pack idea requires further experimentation to understand how social behaviours during predation affect the fitness of single cells and to determine whether predation is a cooperative behaviour or reflects the synergy of individual cells acting side by side [45].

Another striking group behaviour is rippling. This behaviour is seen as macroscopic wave patterns, arising from a swarm of cells that have coordinated cell reversals, and presumably increases the contact time of *M. xanthus* with its prey [6, 44, 46]. Rippling also occurs in response to certain biomolecules, such as peptidoglycan from lysed bacteria, and requires the regulatory activity of a chemosensory two-component system, the Frz pathway [6, 46, 47]. However, it is unknown how a signal is perceived and propagated among single cells to coordinate motility. Rippling can also be observed during development in some strains, presumably caused by autolysis of a subpopulation of *M. xanthus* cells [6].

## Regulation of predation and establishing a reciprocal predator–prey relationship

Predation-specific mechanisms, such as the ATP-dependent protein translocation by the Kil/T3SS* complexes or production of digestive enzymes, are energy intensive, raising the question to what extent are these processes regulated in response to prey. Transcriptome analysis indicates an induction of Kil gene expression upon starvation, but no further increase in the presence of prey [16, 48]. However, expression of several hydrolytic enzymes appears to be upregulated by prey cells, although further studies are needed [49, 50]. Interestingly, *M. xanthus* engaging in predation appear to adapt their lipid metabolism by using alternative pathways for fatty acid and lipid biosynthesis, which might culminate in an altered membrane composition [49]. Upregulation of the mevalonate pathway for isoprenoid synthesis, for example, appears to be a common and preferred pathway, as suggested by comparative genome analysis of predatory and non-predatory bacteria [51]. Transcriptional changes in myxobacteria were also observed in response to isolated acylhomoserine lactones (AHLs), which are released by other bacteria as quorum sensing signals [52, 53]. However, there is no direct link of AHL-regulated genes to known prey killing mechanisms, and it remains to be investigated how this potential eavesdropping by the predator might benefit the predation process.

The interaction of myxobacteria with prey goes beyond killing and consumption, and involves the bilateral competition for scarce resources. This is reflected by transcriptional changes in iron uptake, such as the synthesis of siderophores, which were observed for both predator and prey, e.g. co-cultures of *M. xanthus* with *Sinorhizobium meliloti* or *Streptomyces coelicolor* [49, 50, 54]. Moreover, the production of defensive antibiotics is induced in prey in response to predation, e.g. actinorhodin in *Streptomyces coelicolor* [55] or bacillaene in *Bacillus subtilis* [56]. However, biosynthesis of antibiotics myxovirescin and myxoprincomide in *M. xanthus* is not transcriptionally upregulated by prey or in response to AHLs [49, 52]. Additionally, long-term co-culture experiments of *M. xanthus* with *E. coli* revealed co-evolution by both parties [57]. Here, the genes *eatB* of *M. xanthus *, encoding a membrane protein of unknown function, and *ompT* of *E. coli *, encoding an OM protease, were identified as selection hotspots, although their putative role in predation is currently unknown.

## Kin discrimination

KD in myxobacteria refers to their ability to distinguish between close relatives, particularly clonemates, from more distant myxobacteria [58]. Discrimination plays a central role in their transitions from single cell to multicellularity. However, their transitions face a steep challenge: intrusion of nonkin into vegetative swarms or aggregative multicellular fruiting bodies that disrupt cooperative kin interactions. Such exploitation is likely because soil microbial diversity is high, often consisting of tens of thousands of species per gram [59], as well as dozens of disruptive conspecific isolates [60, 61]. During vegetative predation and growth, cheater cells can scavenge nutrients without necessarily contributing to the shared pool of secreted digestive enzymes and secondary metabolites [34], broadly called public goods. During development, exploitation occurs when a subset of genotypes differentiates into mature spores, while the majority of other cells lyse, in perhaps altruistic acts, to provide nutrients for the metabolically costly programme that takes days to unfold [62].

To prevent exploitation and to ensure social groups remain homogenous, myxobacteria use elaborate KD mechanisms to distinguish self from nonself, and to eliminate nonkin (Fig. 1). The effectiveness of these systems is evident when analysing fruiting body assemblies from natural sources, where cells within fruiting bodies are nearly all clonal [63], despite being derived from environments rich in diversity. Importantly, KD often includes intraspecific discrimination; thus, preventing the formation of mosaic fruiting bodies consisting of different genotypes.

KD systems typically work by delivering polymorphic toxins, which leads to cell death of distant relatives, while protecting clonemates because they express cognate immunity factors. These toxin-immunity cassettes are often associated with mobile genetic elements (MGEs) that promote horizontal transfer [64], where the tight linkage between these genes is critical to ensure the new host simultaneously acquires immunity. To provide exquisite levels of discriminatory specificity against related genotypes, myxobacteria contain dozens of toxin-immunity loci that fluidly move within populations and form unique ‘self-identity barcodes’ in different isolates [65].

Myxobacteria contain three known KD systems – outer membrane exchange (OME), the type VI secretion system (T6SS) and the rearrangement hotspot (Rhs) – all of which deliver toxins that act on fellow myxobacteria and, to date, there are no reports these systems act on other bacterial species [66]. While the specificity of KD varies among these systems, the outcomes are the same: cell death. Myxobacteria likely also contain undiscovered KD systems, some of which may work by nonlethal mechanisms [67].

## Outer membrane exchange

**Fig. 3.**
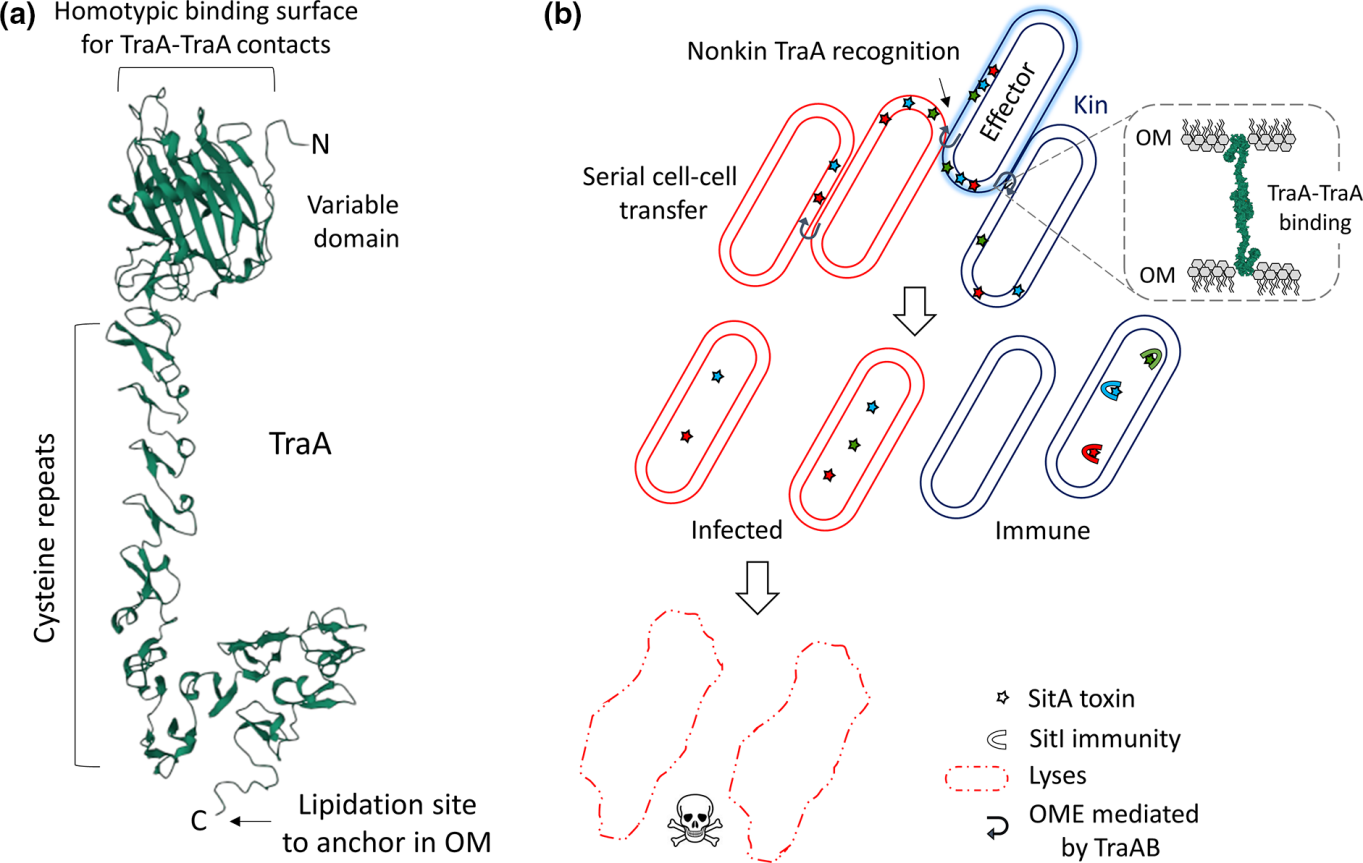
Kin recognition and OME by TraA. (**a**) AlphaFold2 structure prediction of TraA [101], excluding the N-terminal and C-terminal signal peptide and sorting tag, respectively. The variable domain provides specificity for homotypic TraA–TraA binding between cells. Full length proteins contain ~80 cysteine residues where the mature C-terminal residue shown is a cysteine that is predicted to be lipidated, which thus anchors TraA on the cell surface [102]. TraB is an OM β-barrel protein that interacts with TraA and for simplicity is not shown. (**b**) Cells undergoing OME and subsequent consequences. The top centre cell (blue glow) is the effector and when nonkin cells (red) express a TraA compatible receptor, cell poisoning ensues following OME, because they lack immunity to the suite of transferred SitA lipoprotein toxins. Transferred toxins initially reside in the OM of the recipient, where they can subsequently be serially transferred and poison other cells following OME (left red cell). Toxins are eventually transported into the cytoplasm where they act as nucleases in the absence of cognate immunity factors [80]. OME between kin (blue cells) results in no KD. The inset shows an AlphaFold2 prediction of TraA–TraA binding between cells.

OME is a dual functioning platform that mediates KD as well as kin cooperation [68]. It does so through the function of a polymorphic cell surface receptor called TraA and its partner receptor TraB, whose genes overlap in an operon [69]. Self-recognition is mediated by homotypic binding between identical or nearly identical TraA receptors between neighbouring cells; thus, providing highly selective partner recognition (Fig. 3a, b) [68, 70]. TraAB function in cell adhesion, and when overexpressed, cells adhere in end-to-end chains and side-by-side rafts, which alters their motility and social behaviours [71, 72]. In contrast, when adjacent cells express different or incompatible TraA receptors, there is no cell binding nor OME. By using fluorescent fusion reporters, TraAB proteins are found to coalesce into foci upon cell–cell contact and recognition [73], analogous to the Kil and T3SS* complexes described above. Following engagement, OM proteins and lipids are bidirectionally and robustly exchanged between collaborating cells [69, 74]. Although no cytoplasmic proteins nor DNA are exchanged, transferred cargo nevertheless includes hundreds of different OM proteins. Since lipophilic fluorescent dyes and lipopolysaccharides are also transferred [69, 75], the mechanism of exchange is likely mediated by OM fusion where TraAB serve as fusogens.

The outcomes of OME vary widely depending on the nature of partnering cells [68]. As alluded, OME can facilitate cooperation. For example, heterogeneous populations that share and mix cell envelope components via OME transition toward homogeneity, which presumably helps synchronize cooperative behaviours and transition populations toward a tissue-like collective [68, 73]. In other cases, healthy donors transfer and replenish defective components to low fitness siblings resulting in their revitalization [69, 75]. In turn, the healthy cells also benefit by increasing the population size of fit cells to threshold levels for multicellular functions, including development [76].

In cases where cells express compatible TraA receptors, but are not siblings, antagonism ensues by the transfer of a suite of lipoprotein toxins (Fig. 3b) [77]. These effectors are named SitA, for swarm inhibition toxins, because in mixed colony populations between nonkin, outward swarm expansion is blocked by OME-mediated cell poisoning [78, 79]. SitA effectors belong to six distinct families (SitA1/2–SitA7) defined by their unique N-terminal ‘escort domains’ [65]. At the C-terminus, each effector contains a toxin domain that belongs to one of >30 different families, many of which are found in other conflict systems. Interestingly, all the C-terminal domains that are characterized or have predicted functions are nucleases [65, 77]. Consequently, once delivered to the inner leaflet of target cells by OME, the toxin module must traverse the cell envelope to reach the cytoplasm (Fig. 3b). This migration involves a secondary step to OME and is mediated by their escort domains, which facilitate transport across the cytoplasmic membrane [80]. This secondary step of toxin migration is not instant and, consequently, cells become ‘infected’ and can transfer toxins to their siblings by OME (Fig. 3b) [77]. As a result, this KD system is extremely potent where one inhibitor cell can infect >1000 target cells by serial toxin transfer among cells. In contrast, if the recipient cell is clonal, it will contain the cognate suite of SitI immunity proteins and is not harmed.

The number of *sitAI* loci in myxobacterial genomes is large, ranging from 15 to >80, where a single difference can result in lethal OME [65]. However, toxic encounters only occur when strains express compatible TraA receptors, which again are highly polymorphic; thus, guarding against unintentional lethal exchanges with distant relatives. Nevertheless, since the number of functional TraA polymorphisms is finite, lethal encounters occur. Moreover, unique *sitAI* loci are rapidly disseminated through populations by their association with MGEs, resulting in KD between recent siblings. With this knowledge, comparative genomics serves as a viable approach to predict OME social compatibilities among natural isolates based on their *sitAI* and *traA* allele composition [65, 66]. Finally, because OME is a mutual decision, where both cells must express compatible TraA receptors to share their private cellular goods, we hypothesize OME originally evolved as a cooperative mechanism that subsequently was co-opted for KD [68].

Genomic analysis of *sitAI* loci frequently reveals they reside on a prophage, plasmid or transposons [65, 66, 77]. These and other findings led to our conclusion that MGEs exploited OME as a means for their expansion and retention in populations. For instance, when a cell acquires a selfish element with a unique *sitAI* locus, that transformed cell can now kill and outcompete its siblings by cell–cell intoxication [77, 80]. Additionally, if the element is lost by excision or deletion, then that cell is susceptible to poisoning by neighbouring siblings. As revealed by genomics, the benefits of *sitAI* loci outweighs their loss, explaining their large numbers in genomes, even though they represent a significant genomic burden (e.g. 100 kb of DNA). In turn, along with the immediate host benefiting from acquired selfish elements, populations at large may also benefit because new *sitAI* loci provide renewed policing mechanisms to guard against exploitation of their cooperative interactions [64]. Finally, we propose the evolutionary forces that drive *traA* diversification and maintenance of its polymorphism were done so to select against lethal OME encounters between nonkin. In addition, since OME plays dual roles in both cooperation and KD, this explanation provides a solution to Crozier’s paradox, namely how cooperative genes can be polymorphic [77, 81].

## T6SS

The T6SS is a multicomponent protein secretion system that is widely distributed amongst Gram-negative bacteria and is thought to be evolutionarily related to contractile phage tails [82]. It punctures target cell envelopes and delivers effectors, which are often associated with the VgrG or PAAR spike proteins or the Hcp tube. T6SS effectors act on the cell wall, membrane, nucleic acids or other essential components [83]. T6SS is frequently used to attack other bacteria and in some cases eukaryotic cells. In *M. xanthus *, there is one T6SS gene cluster composed of 14 genes that encode the apparatus. In contrast, effector-immunity loci are scattered around the chromosome. Similar to *sitAI* loci, many T6SS effector-immunity cassettes are found on prophage, particularly a family called Mx-alpha. In turn, these MGEs enable rapid ecological dissemination that results in social diversification of populations [64, 66]. Cryo-electron microscopy revealed expanded and contracted forms of the T6SS apparatus in *M. xanthus* cells, as well as associated cell surface filaments or ‘antenna’, which may be involved in target cell recognition and, if so, work independently of TraA recognition [84].

Because of their predatory behaviours and the widespread use of T6SS as microbial weapons, it was reasoned myxobacteria use them in bacteria predation. However, studies by different labs found no evidence for this role [16, 17, 41]. Instead, T6SS is primarily involved in social policing or KD. This finding was first made by Troselj *et al.*, who found that phenotypically less fit cells were killed by more fit siblings [85]. Experimentally, this was shown with isogenic strains where prototrophs killed their auxotroph siblings via the T6SS. However, antagonism only occurred when an essential metabolite, e.g. histidine for auxotrophs, was absent from media, thus blocking their growth, while prototrophs grew in mixed colonies. Interestingly, the responsible T6SS effector-immunity locus, *tsxEI*, is conserved within the genus *Myxococcus *, unlike other T6SS polymorphic effectors, suggesting a conserved mechanism for social policing against less fit siblings. Although the auxotroph strains contain the identical *tsxEI* locus, their sensitivity to intoxication occurs because during metabolite starvation the level of the TsxI immunity protein decreases with concurrent loss of protection to TsxE.

In separate work, Li and colleagues used a genetic screen for swarm incompatibility to identify *M. xanthus* mutants that exhibited KD by the parent strain [86]. They identified mutations in six gene clusters, encoding T6SS toxin-immunity cassettes, which resulted in cell death when mixed with the parent strain [87]. One of the toxins encodes a nuclease that when expressed from a plasmid kills the *E. coli* host, while co-expression of the downstream immunity gene neutralizes toxicity [88].

In another study, involving 22 sympatric *M. xanthus* soil isolates, the effect of T6SS and OME on KD was assessed. Here, for strains containing compatible TraA receptors, it was necessary to create double mutants to relieve antagonism; thus, OME and T6SS act redundantly in KD. However, for two isolates antagonism remained and involved a third system (see below) [66]. Strikingly, the discriminatory T6SS effectors were located on prophage Mx-alpha, as well as many of the OME effectors. Consistent with these findings, T6SS are also involved in KD in other genera, such as *Vibrio* [89, 90] and *Proteus* [91, 92]. In summary, T6SS provides a broad-spectrum KD mechanism in myxobacteria, while OME provides a narrow-spectrum KD mechanism based on TraA compatibility.

Finally, in a recent study, a specific T6SS toxin showed anti-fungal activity, but was not active in KD when a partner cell lacked the cognate immunity gene [87]. To date, this is the only known case in *M. xanthus* where T6SS is involved in inter-species competition or predation. This protein, MXAN_1813, contains a PAAR domain, such domains are frequently associated with T6SS toxins, and a C-terminal domain of unknown function that is only found in myxobacteria. Because of this finding, it is possible other T6SS effectors may function, in a limited sense, in predation.

## Rhs proteins

Rhs proteins are widely found in bacteria, contain YDxxGRL(I/T) repeat sequences related to YD-repeats and C-terminal polymorphic toxin domains [93]. Rhs proteins can be divided into two broad classifications, where one group are very large proteins, usually >1500 amino acids, contain signal peptides and likely form filamentous cell surface appendages. In contrast, the second group, typically found in Gram-negative bacteria, are much smaller proteins, e.g. several hundred amino acids, lack signal peptides and are frequently delivered by T6SS. Many of the characterized Rhs proteins function in intra- and inter-species antagonism [94, 95], such as WapA protein of *B. subtilis *. Additionally, some Rhs proteins are virulence factors in pathogenic organisms [96].

In the above study of 22 *M. xanthus* soil isolates, nine large *rhs* genes were identified from genomic sequences. Seven of these genes were conserved among clade members, while two *rhs* genes (*rhs4* and *5*), each encoding ~4000 amino acids, were unique and shown to be involved in KD [60, 66]. Their role in KD was discovered after the OME and T6S systems were inactivated and antagonism persisted. Thus, antagonism was relieved only after knocking out these *rhs* genes, as well as OME and T6SS [66]. Strikingly, in contrast to their other *rhs* genes, *rhs4* and *5* are located on different Mx-alpha prophage, as are unique OME and T6SS effectors. Moreover, since antagonism remained in T6SS mutants, the delivery of Rhs4 and Rhs5 is not T6SS dependent. These Rhs proteins contain signal sequences and likely reside on the cell surface where they bind and deliver C-terminal toxins to neighbouring cells [66], presumably analogous to contact-dependent growth inhibition (CDI) toxin delivery [2]. In support of this, an Rhs protein from *Pseudomonas aeruginosa* localizes on the cell surface where it is involved in virulence [96]. Whether *M. xanthus* Rhs proteins function in interspecies competition remains an open question.

While there is no evidence that myxobacteria use Rhs proteins for predation, one study found they play an inhibitory role. Here, a transposon insertion in the *rhs* gene (MXAN_6679) resulted in a gain-of-function or hyper-predation phenotype on *B. subtilis* [41]. In a separate genetic screen, a MXAN_6679 insertion mutant resulted in a motility defect [97]. Clearly, further studies are needed to understand the roles Rhs proteins play in myxobacterial social interactions.

## Concluding remarks

**Fig. 4.**
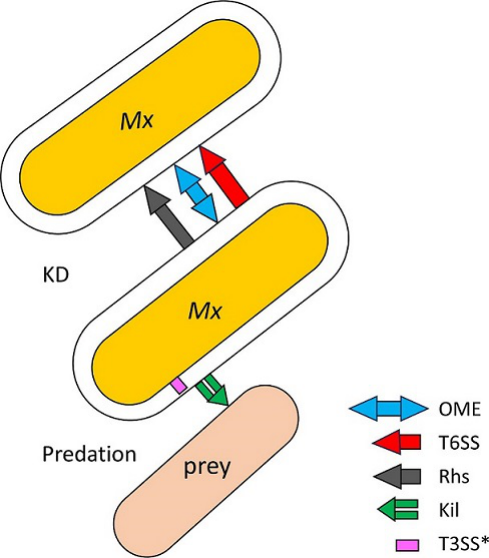
Conflict systems mediated by cell–cell contact in *M. xanthus* (*Mx*) and their biological roles.

Predation and KD serve central but different roles in the lifecycles of myxobacteria (Fig. 1). However, both functions rely on conflict systems that recognize target cells to initiate effector transfer resulting in target cell death (Fig. 4). Recent efforts by different groups have revealed important insights of the molecular basis of these systems; but one remaining question is, to what extent predation and KD might overlap. Theoretically, the TraA receptor or a similar polymorphic marker could also define the target spectrum for predation, e.g. by blocking the assembly of the Kil/T3SS* machinery between kin via a, yet to be identified, signalling mechanism. Given the very broad prey spectrum of myxobacteria, such a protective or exclusion mechanism might be more likely than the active recognition of different structures on prey cells. Additionally, the killing factors released by the Kil/T3SS* machinery are unknown, but might be similar or possibly identical to the toxin-immunity pairs used during KD, and/or involve separate effector classes, such as digestive enzymes. Finally, the ability to use one or more of the described KD systems to kill siblings might lead to cannibalistic behaviours, such as those reported in *B. subtilis* and suggested in other species [98–100]. Although killing by related myxobacterial occurs, to date there are no reports that the killer acquires nutrients that supports measurable growth as found against distant prey species. It is clear at this point, however, that with their conflict systems the social, yet predatory, myxobacteria have established an efficient way to manage the manifold interactions with other bacteria to their advantage.
